# Single cell RNA sequencing reveals a dysbalance of proinflammatory vs. immunosuppressive dendritic cells in mouse and human aortic aneurysms

**DOI:** 10.3389/fcvm.2025.1713030

**Published:** 2025-11-19

**Authors:** Yi Ran, Jingpu Zhu, Ting Sun, Yixin Zhang, Chuankai Zhang, Yutao Li, Zhipeng Li, Shu Wang, Liping Li, Junjie Zheng, Changjun Yin, Andreas J. R. Habenicht, Zhihua Wang

**Affiliations:** 1 Institution of Precision Medicine, The First Affiliated Hospital of Sun Yat-sen University, Guangzhou, China; 2 Division of Vascular Surgery, The First Affiliated Hospital of Sun Yat-sen University, Guangzhou, China; 3 Institute for Cardiovascular Prevention (IPEK), Ludwig-Maximilians-University (LMU), Munich, Germany; 4 Department of Oncology, Cancer Center, The First Affiliated Hospital of Sun Yat-sen University, Guangzhou, China; 5 DZHK (German Centre for Cardiovascular Research), Partner Site Munich Heart Alliance, Munich, Germany; 6 Easemedcontrol R&D, München, Germany; 7 NHC Key Laboratory of Assisted Circulation and Vascular Diseases, Sun Yat-sen University, Guangzhou, China; 8 Nanjing Key Laboratory for Cardiovascular Information and Health Engineering Medicine, Cardiovascular Medical Center, Medical School, Institute of Clinical Medicine, Nanjing Drum Tower Hospital, Nanjing University, Nanjing, China

**Keywords:** aortic aneurysm, dendritic cells, inflammation, immunosuppression, dysbalance

## Abstract

Immune cell-driven destruction of the aortic wall remains a major contributor of death in patients burdened with aortic aneurysms (AAs). Dendritic cells (DCs) play critical roles in bridging innate and adaptive immunity by orchestrating robust inflammatory responses and concomitantly sustaining immune tolerance. However, the specific roles of DCs in AA pathogenesis remain to be explored. To examine the participation of DCs in AA pathogenesis, we used single-cell RNA sequencing (scRNA-seq) integration analyses to characterize DC heterogeneity and elucidate their putative involvement in AA pathogenesis in several mouse AA models and translate the experimental data to human AAs. Our data reveal that conventional DC2s (cDC2s) constituted the most abundant DC subtypes in both murine and human AAs. Furthermore, cDC1s, plasmacytoid DCs (pDCs) and immunosuppressive mature regulatory DCs (mregDCs) were identified. Within the cDC2 subtypes, the AA tissue environment trained cDC2s and a newly defined DC3s subtype toward highly pro-inflammatory phenotypes. Parallel to the increased prevalence of pro-inflammatory activated cDC2s and DC3s, a significant reduction of the number of mregDCs was observed in mouse AAs. This data revealed that the balance between pro- vs. the anti-inflammatory DCs is disrupted in mouse AAs. Thus, therapeutic reconstitution strategies to correct this dysbalance together with protective measures that are already in use in clinical practice may lead to beneficial AA outcomes before surgical intervention is needed.

## 1 Introduction

AAs account for approximately 170,000 annual deaths worldwide, representing a significant contributor to global cardiovascular disease mortality (1, 2). Multiple genetic and environmental risk factors contribute to the development of AAs including atherosclerosis, hypertension, smoking, age, gender, infectious diseases and rare monogenetic mutations of the Marfan Syndrome (1–4). Various intervention approaches have been developed with not only a prominent role of blood pressure lowering drugs but also mast cell inhibitors, and anti-platelet drugs, have been applied (1). However, none of these treatments achieved beneficial effects to reduce AA mortality in randomized placebo-controlled trials (5–8). Surgical approaches including stent implantation and aorta segment replacement remain standard procedures under conditions of unmanageable AA progression (9). Therefore, research into preventive AA treatment options remains an important unmet medical need.

The majority of AAs involves complex cellular and molecular mechanisms that drive disease initiation and progression. Smooth muscle cells (SMCs) play central roles in AA pathogenesis and SMC phenotype switching and cell death pathways have been identified as major contributors to exacerbate the progression of AAs (3). Immune cell infiltration and inflammation-driven destruction of aortic walls also contribute and T cells, macrophages, neutrophils and NKs have been identified as culprits of AA progression (3). Among these, CD4^+^ T cells and macrophages are the most abundant immune cell lineages in human AAs (10). Experimental studies in mouse models demonstrated that the absence of CD4^+^ T cells confers resistance to AA development (11). These cells promote the development of AAs via IL-1β, IL-6, and TNF-α, several chemokines and matrix metalloproteinases forming a life-threatening cocktail to advance arterial wall destruction (3, 12, 13). In contrast, the role of DCs in AA pathogenesis remains unknown (14). DCs are broadly categorized into plasmacytoid DCs (pDCs), monocyte-derived DCs (moDCs), and conventional DCs (cDCs) (15). cDCs are further subdivided into cDC1s (specialized in cross-presentation to CD8^+^ T cells) and cDC2s (primarily activating CD4^+^ T cells) (16). Recent advances in scRNA-seq have established further DC subtypes in both mice and humans (17–20) and their impacts in cancer growth, autoimmune diseases and multiple chronic inflammatory diseases are being studied (15, 21–24). Here, building on this recent progress in other diseases, we adopted a multipronged strategy including multiple scRNA-seq datasets and pan-species integration analyses to analyze the DC landscape across multiple AA mouse models and human phenotypes of AAs. Our data revealed a pronounced shift in DC subsets in AAs vs. control mice and normal donor aortas, respectively, with an increase in pro-inflammatory and markedly activated cDC2s and DC3s concomitant with a numerical decline in anti-inflammatory immunosuppressive mregDCs. We suggest that this dysbalance of DC subsets may participate in the progression of AAs and that reconstitution strategies to target DCs may be beneficial to delay surgical intervention.

## 2 Results

### 2.1 scRNA-seq indicates participation of DCs in the pathogenesis of mouse AAs

**Figure 1:**
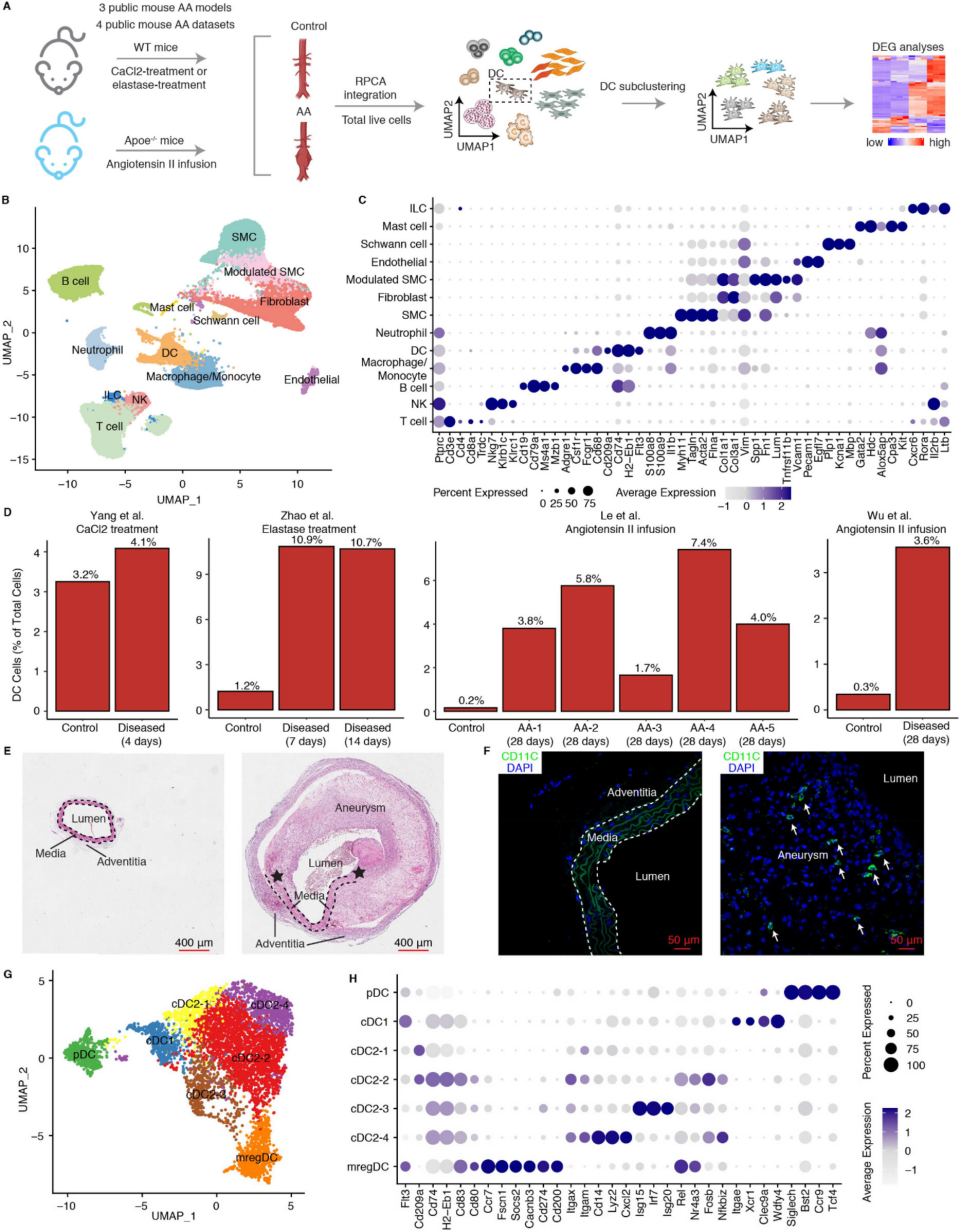
ScRNA-Seq characterizes the phenotype and changes of DCs in mouse AAs. **(A)** Schematic workflow shows the scRNA-seq integration approach in mouse AAs. Three public mouse models from four public datasets were used. WT, wild type; RPCA, reciprocal principal component analysis; DEG, differentially expressed genes. **(B)** UMAP visualization of defined cell types in mouse AAs and controls. **(C)** Dotplot shows the cell type lineage-specific gene markers to define different cell types. **(D)** Changes in DC percentages across four different mouse AA studies. AA-1 to AA-5 in Le et al. study represent different types of mouse AAs. AA-1, abdominal dilated aorta without dissection; AA-2, single aortic abdominal aneurysm without dissection; AA-3, abdominal aortic dissection/intramural haematoma without aneurysm; AA-4, single abdominal aortic aneurysm with dissection/intramural haematoma; AA-5, multiple distinct abdominal aortic aneurysms with dissection/intramural haematomas. Representative H&E stainings **(E)** and fluorescent immunostaining for CD11C **(F)** of mouse abdominal aortas from control mice (left panel) and Ang-II induced AA mice (right panel). Arrows indicate DCs (CD11C^+^). Asterisks represent the disrupted elastic fibers. **(G)** UMAP visualization of the DC subtypes in mouse AAs and controls. **(H)** Dotplot displays cell type-specific or highly expressed signature genes in each DC cluster. SMC, smooth muscle cell; NK, natural killer cell; ILC, innate lymphoid cell.

We first used an integrated multi-model scRNA-seq analysis integration strategy across three established mouse models, including the Calcium chloride- (CaCl2) induced, the elastase-induced, and the angiotensin II-induced models (25–28) (Figure 1A; Supplementary Table S1). Low quality cells and doublets were excluded resulting in a total of 88,324 high-quality cells for further analysis (Figure 1B). Cells were classified into 12 distinct subtypes based on their canonical cell type-specific markers, comprising 5 non-immune cell types (including SMCs, fibroblasts, endothelial cells, modulated-SMCs, Schwann cells) and 7 immune cell types (macrophages, neutrophils, DCs, B cells, T cells, NK cells and innate lymphoid cells) (Figure 1C). SMCs and fibroblasts were the predominant cell types in the control groups (Supplementary Extended Figure 1). In contrast, AA tissues across all models exhibited a relative decline of these cell types with compensated relative increases of immune cells. Notably, the CaCl2 model showed a relatively higher increase in neutrophils and macrophages whereas the elastase-and angiotensin II-induced models showed elevated levels of both adaptive and innate immune cells, suggesting differences in the participation of innate vs. adaptive immune cells across mouse models (Supplementary Extended Figure 1). Moreover, DCs constituted only 0.2%–1.2% of cells in healthy aortas of most studies, yet exhibited an over 8-fold increase in proportion in AAs across different elastase-and angiotensin II-induced models (Figure 1D), suggesting a significant role in AA formation. We further examined Ang-II induced AAs to assess DCs in AA lesions by immunohistochemical studies: We observed a significant accumulation of DCs in the region of aneurysm compared to the intact aortas of control mice (Figures 1E,F).

To detail the composition and heterogeneity of DCs across different AA mouse models, we extracted 5,522 DCs for subtype characterization from scRNA-seq data (Figure 1G) as described in methods. Anchor-based reciprocal principal component analysis (RPCA), which is superior in multiple sample integration and batch effect correction algorithms (29), was employed for this purpose. The sample distribution in the UMAP visualization along with the significantly elevated integration local inverse Simpson's indices (iLISI) and significantly decreased cell type LISI (cLISI) values after batch effect correction confirmed robust data integration across multiple datasets (Supplementary Extended Figures 2A–D). DCs were classified into 7 cell subtypes (Figure 1G). Consistent with our previous findings in atherosclerosis (21), which identified the presence of pDCs, cDC1s, cDC2s and mregDCs. The mouse AAs also revealed the presence of cDC1s characterized by significant expression of *Itgae*, *Xcr1*, *Clec9a* and *Wdfy4*, while pDCs exhibited elevated expression of *Siglech*, *Cst2*, *Ccr9* and *Tcf4* (Figure 1H). We identified 4 cDC2 clusters which we provisionally designated as cDC2-1, cDC2-2, cDC2-3, and cDC2-4, all of which showed high expression of *Itgam* and *Itgax* (Figure 1H). The mregDCs (mature DCs enriched in immunoregulatory molecules) distinguished themselves from other DC subtypes by significant expression of co-stimulatory genes (*Cd80*, *Cd83*), migration-related genes (*Ccr7*, *Fscn1*, *Socs2*, *Cacnb3*) and particularly co-inhibitory related genes (*Cd274*, *Cd200*) (Figure 1H).

### 2.2 scRNA-seq identifies pro-inflammatory activated cDC2s and DC3s in mouse AAs

**Figure 2:**
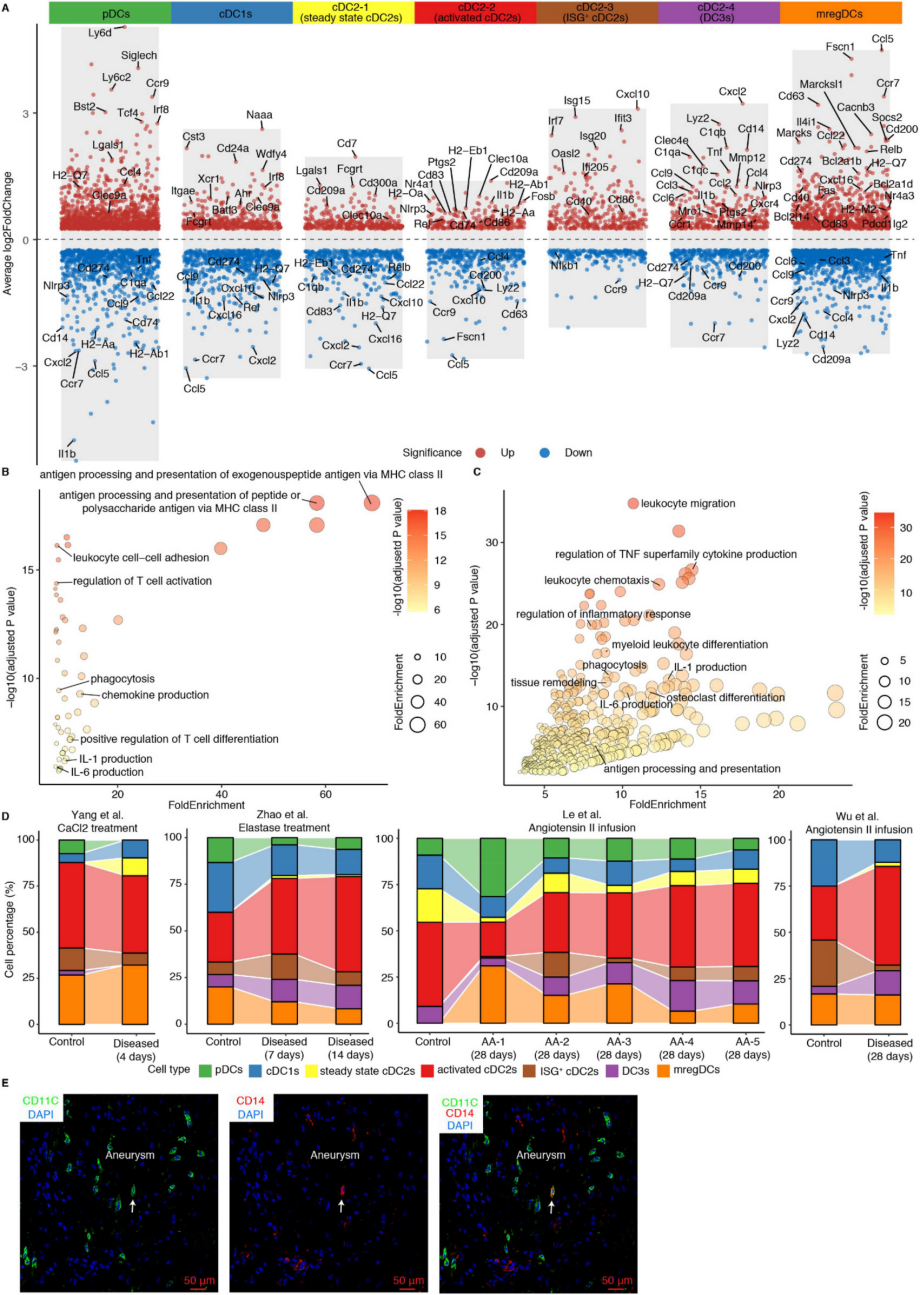
Phenotypes of DC subtypes in mouse AAs. **(A)** Volcano plot illustrates the cell type-specific and highly expressed genes across different DC subtypes. Pathway enrichment analyses show significantly enriched pathways in activated cDC2s **(B)** and DC3s **(C)**. **(D)** Cell composition of DC subtypes across 4 different mouse AA studies. AA-1 to AA-5 in Le et al. study represent different types of mouse AAs. AA-1, abdominal dilated aorta without dissection; AA-2, single aortic abdominal aneurysm without dissection; AA-3, abdominal aortic dissection/intramural haematoma without aneurysm; AA-4, single abdominal aortic aneurysm with dissection/intramural haematoma; AA-5, multiple distinct abdominal aortic aneurysms with dissection/intramural haematomas. **(E)** Representative images of DC3 (CD14^+^CD11C^+^) in Ang-II-induced mouse AA. Arrows indicate CD14^+^CD11C^+^ DC3.

The heterogeneity of cDC2s has been well-documented (18, 30). To characterize the gene expression profiles of distinct DC subtypes in AAs, we performed differentially expressed gene (DEGs) analyses across DC subtypes (Figure 2A). We observed 4 different cDC2 subtypes: cDC2-2 showed significantly elevated expression of genes associated with cell activation, including *Rel*, *Nr4a3*, *Nfkbiz*, and *Fosb*, suggesting an activated cDC2 phenotype. Inflammation-associated genes such as *Il1b*, *Nlrp3*, and *Ptgs2* were enriched in this cluster, further supporting its pro-inflammatory role (Figure 2A). Interestingly, the cDC2-3 subset was characterized by higher expression of interferon-related genes, including *Isg15*, *Irf7* and *Isg20*, defining an interferon-stimulated gene-expressing cDC2 (ISG^+^ cDC2) subtype. Unlike other cDC2 subtypes, the cDC2-4 subset revealed a unique transcriptional profile with high expression of monocyte-related genes (*Cd14*, *Lyz2*, *Cxcl2*) together with inflammation- and complement pathway-associated genes (*C1qa*, *Ccl2*, *Tnf*, *Mmp12*, *Nlrp3*, *Cxcl16*, *Ptgs2*) (Figure 2A). This gene signature shows resemblance with the newly defined DC3 cell subtype in mice revealing characteristics of both cDC2s and monocytes but its origin most likely emerges from Ly6C^+^ monocyte-DC progenitors (MDPs) as indicated by the work of others (18). Conversely, the cDC2-1 subset lacked significant expression of activation-related genes indicating a steady state or quiescent cDC2 cell population (Figure 2A). As professional antigen presenting cells, activated cDC2s showed significant enrichment in pathways related to antigen processing and presentation, cell adhesion, T cell activation, phagocytosis, and inflammation responses associated with IL-1 and IL-6 production, suggesting their critical role in directing inflammation in mouse AAs (Figure 2B). Of note, consistent with the high expression of inflammation-related genes, DC3s showed an even more pronounced enrichment in the inflammatory response pathway. Pathways associated with inflammatory response, such as TNF superfamily cytokine, IL-1 and IL-6 production, leukocyte migration and phagocytosis were significantly enriched in the DC3s, indicating their pro-inflammatory phenotype. Interestingly, the tissue remodeling and osteoclast differentiation pathways were also significantly upregulated in DC3s, indicating their potential role in contributing to aortic remodeling in mouse AAs (Figure 2C). In contrast, ISG^+^ cDC2s and steady state cDC2s did not show strong inflammatory-associated pathway gene expression patterns (Supplementary Extended Figures 3A,B). More importantly, cellular composition revealed that both activated cDC2s and DC3s cell populations were elevated in elastase and angiotensin II-induced mouse AA models (Figure 2D). Immunofluorescent staining also showed the presence of DC3s (CD14^+^CD11C^+^) in mouse AAs (Figure 2E).

To elucidate the specific phenotypes of activated cDC2s and DC3s in AAs vs. their counterparts in control arteries, we observed elevated expression of *Csf1r* in cDC2s and DC3s (Supplementary Extended Figure 4A). Previous studies had established that *Csf1r* mediates the expansion and differentiation of DCs (31). Analysis of *Csf1r* ligands revealed broad expression of *Csf1* among fibroblasts, SMCs, modulated SMCs, mast cells, and neutrophils, whereas *Il34* expression was undetectable in mouse AAs (Supplementary Extended Data Figure 4B). Notably, the upregulated expression of *Csf1* and the significantly predicted CSF1-CSF1R interaction in mouse AAs compared to controls suggests that the diseased microenvironment in mouse AAs may promote the expansion and differentiation of activated cDC2s and DC3s through the CSF1-CSF1R signaling axis (Supplementary Extended Data Figures 4C,D). Coupled with the substantial increase of DC abundance in AAs, all these findings indicate that the increase of activated cDC2s and DC3s, along with their trained pro-inflammatory properties may contribute to AA formation.

### 2.3 Gene profiles of mregDCs in AAs indicates their anti-inflammatory and immunosuppressive roles in AA pathogenesis

**Figure 3:**
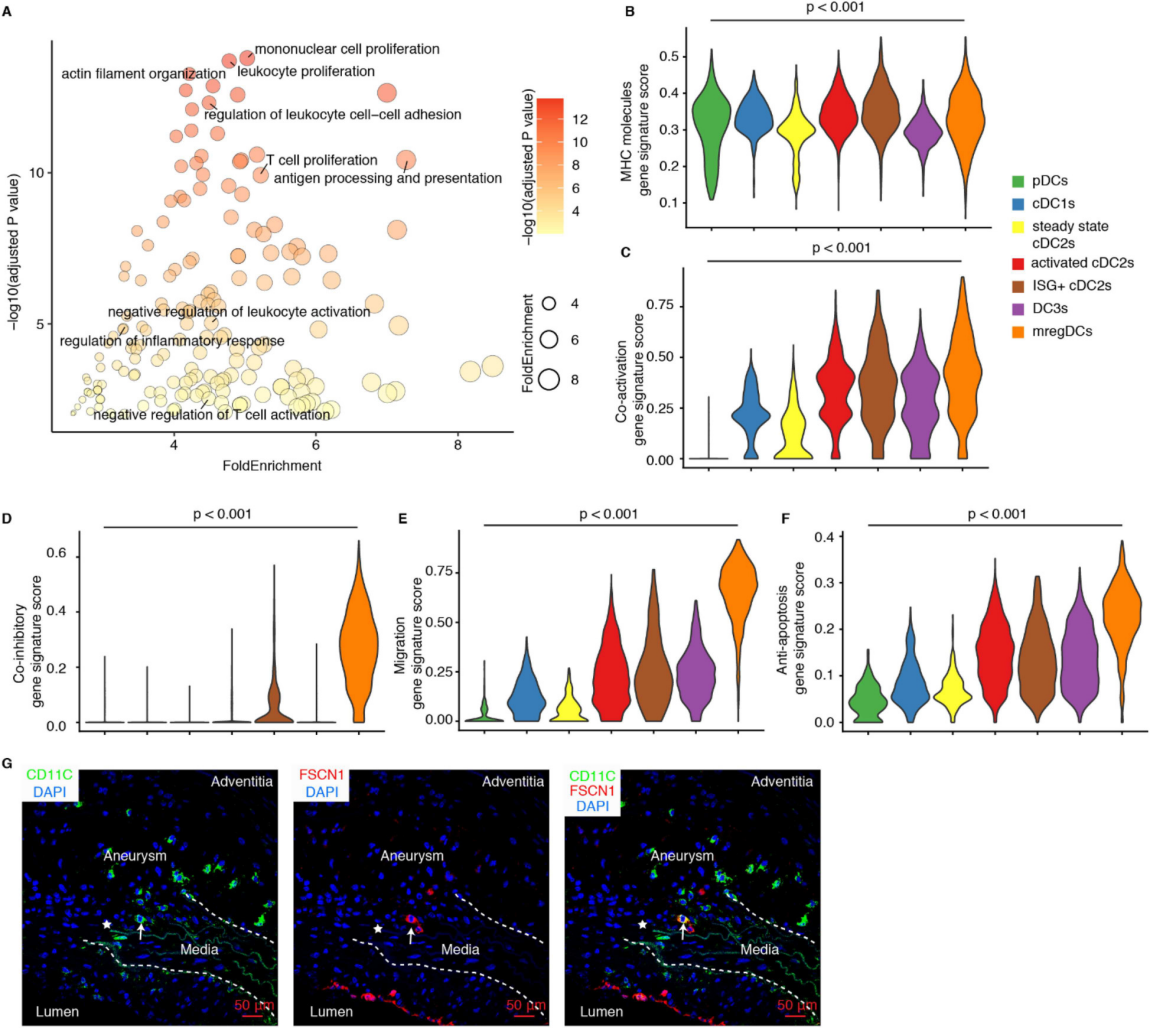
Phenotype and potential function of mregDCs in mouse AAs. **(A)** Pathway enrichment analyses show significantly enriched pathways in mouse mregDCs. Gene signature scores characterize the capacity of MHC molecules **(B)**, co-activation **(C)**, co-inhibition **(D)**, migration **(E)** and anti-apoptosis **(F)** across different DC subtypes in mouse AAs. Kruskal–Wallis rank sum test was used to compare the differences among different DC subsets. **(G)** Immunofluorescence staining reveals the presence and distribution of mregDCs (FSCN1^+^ CD11C^+^) in Ang-II-induced mouse AAs. Arrows indicate FSCN1^+^ CD11C^+^ mregDC in AA lesions. Asterisks represent the disrupted elastic fibers.

The immunosuppressive function of mregDCs has been extensively documented in various cancers, where they exacerbate tumor progression by inhibiting effector CD4/CD8T cells while promoting Treg cell functions (19, 32), but their regulatory functions in AA pathogenesis has not been studied. Moreover, pathway enrichment analyses revealed their multifaceted roles in both immune cell activation and negative regulation of immune system in mregDCs in AAs (Figure 3A). Significant expression of *Cd80* and *Cd83* confirmed the mature phenotype of mregDCs in AAs, while elevated levels of *Ccr7*, *Fscn1* and *Cacnb3* point to their robust migratory capacity, enabling them to trigger adaptive immune responses and migrate from diseased sites to secondary lymphoid organs (Figure 2A). DCs (with the exception of pDCs) are the primary and most specialized immune cell lineage to initiate adaptive T cell immune responses, owing to their superior abilities to promote antigen presentation, co-stimulation, and migration to lymph nodes (14, 33). To delineate potential roles of mregDCs vs. other DCs, we first evaluated their antigen-presenting capacity gene profile, as reflected by MHC expression levels. Significant differences in MHC molecules module scores across different DC subtypes became apparent (Figure 3B). MregDCs showed compatible MHC molecules module scores with activated cDC2s, ISG^+^ cDC2s, cDC1s, and pDCs, whereas steady state/quiescent cDC2s and DC3s subtypes showed lower MHC expression levels (Figure 3B). Furthermore, mregDCs, activated cDC2s, ISG^+^ cDC2s, DC3s cells all exhibited stronger immune activation potential vs. steady state cDC2s, cDC1s and pDCs (Figure 3C). Notably, mregDCs were the predominant population exhibiting robust immunosuppressive function-related genes (Figure 3D). The migratory capacity is critical for initiating immune responses in remote lymph nodes. The migration score varied among different DC subtypes, with mregDCs achieving the highest levels (Figure 3E). These analyses support the anti-inflammatory role of mregDCs in mouse AAs. Importantly, mregDCs abundance declined with disease progression revealing a potential impactful dysbalance between AA-promoting vs. AA-attenuating DC subsets in elastase-induced and angiotensin II-induced AA mouse models (Figure 2D). Additionally, anti-apoptotic genes, including *Bcl2a1b*, *Bcl2a1d*, *Fas*, and *Bcl2l14*, were significantly upregulated in mregDCs. Indeed, the anti-apoptosis scores were markedly elevated in mregDCs, activated cDC2s, ISG^+^ cDC2s and DC3s, with mregDCs exhibiting the most pronounced anti-apoptotic capacity potential of all DC subsets (Figure 3F). By utilizing the specific and highly expressed gene *Fscn1*, we identified the presence of mregDC (FSCN1^+^CD11C^+^) in AA lesions (Figure 3G). This data strongly supports the presence and impact of roles of mregDCs in AAs.

### 2.4 Pronounced heterogeneity of cDC2s and the AA-induced pro-inflammatory differentiation of DC3s

**Figure 4:**
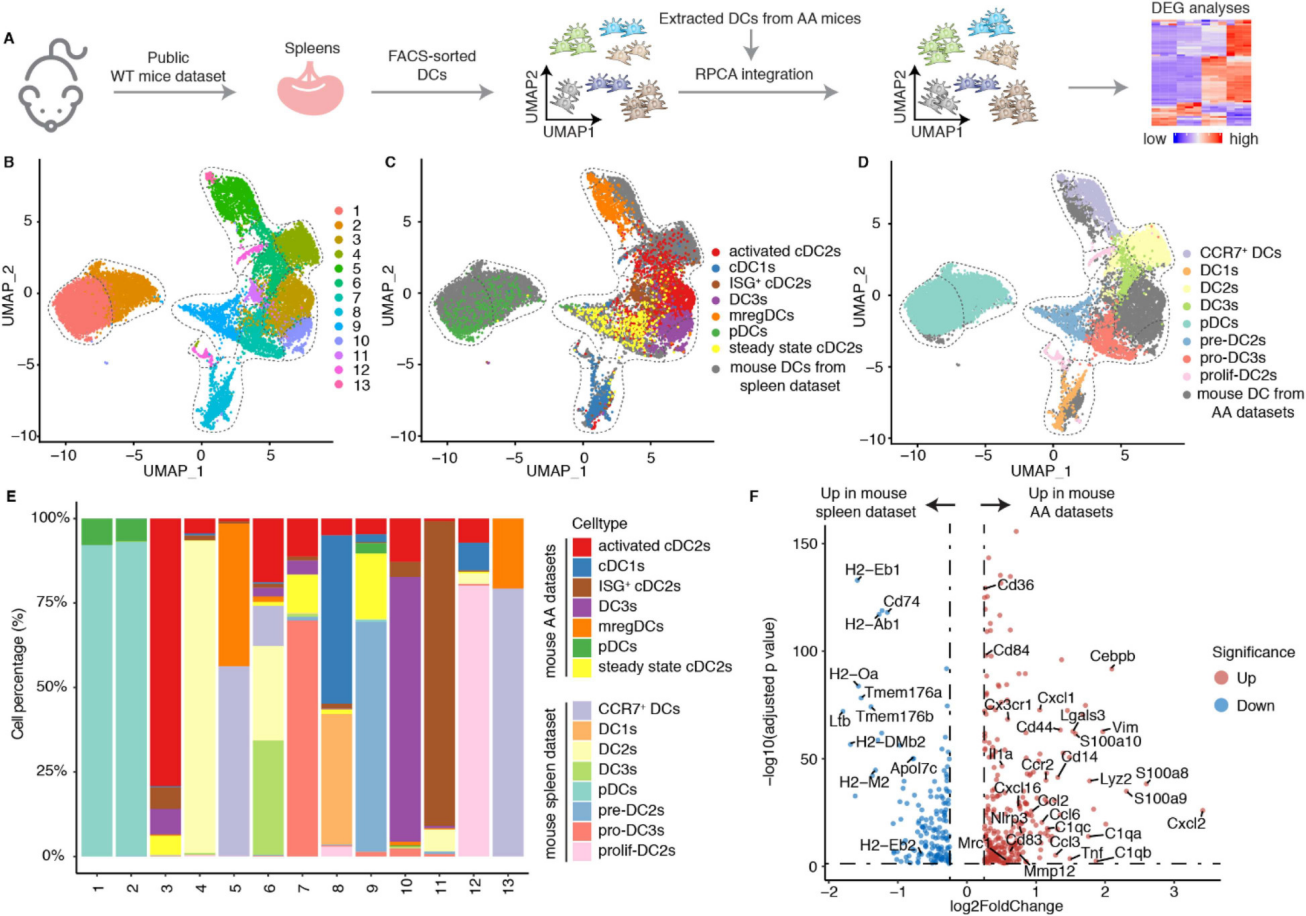
The mouse AA tissue environment reshapes the phenotype of DC3s. **(A)** Publicly available FACS-sorted splenic DC scRNA-seq data was integrated into the DC data of mouse AAs. **(B)** UMAP visualization the cell distribution of different DC clusters from mouse AA and spleen datasets. **(C)** UMAP visualization shows the distribution of different DC subtypes in mouse AA datasets. **(D)** UMAP visualization shows the distribution of well-defined DC subtypes in mouse spleen datasets. **(E)** The cell percentages of each cluster by different DC subtypes from mouse AAs and spleen datasets. **(F)** Volcano plot displays the differentially expressed genes of DC3s between mouse AA and spleen datasets.

The gene expression profiles of different DC subtypes have been well-documented in the spleen (18). We integrated a publicly available scRNA-seq dataset from spleen of Cx3cr1^GFP^ mice (C57BL/6J background) with DCs from the mouse AA datasets (18) (Figures 4A,B). The spleen dataset defined multiple DC subtypes and was used as a positive control. Our integration data revealed that mregDCs, pDCs, and cDC1s from spleen and mouse AA samples clustered together indicating similarities between spleen and AA DC subtypes (Figures 4C,D). In contrast, cDC2s exhibited marked heterogeneity, consistent with previous reports (18, 30). We calculated the proportional contribution of predefined cell types from each dataset to assess their representation and overlap (Figure 4E). Clusters 1 and 2 were predominantly composed of pDCs, clusters 5 and 13 of mregDCs, and cluster 8 of cDC1s from both spleen and AA datasets, reflecting their transcriptional similarity (Figure 4E). Activated cDC2s, ISG^+^ cDC2s and DC3s from the mouse AA datasets primarily contributed to a distinct cluster, revealing significant dissimilarities of cDC2 profiles between hemostatic and diseased conditions indicating that the AA immune environment trains cDC2s differentiation into various subtypes. We next analyzed DEGs across different DC subtypes between homeostatic spleen and diseased mouse AA tissues. In contrast to the undisturbed spleen DCs, DC3s in the AA microenvironment exhibited upregulated expression of genes associated with activation, migration, inflammation, and antigen presentation (Figure 4F). However, cDC1s, mregDCs and pDCs showed only minor transcriptional changes (Supplementary Extended Figures 5A–C). Collectively, these findings confirm the pronounced heterogeneity of cDC2s and demonstrate that the AA immune microenvironment enhances DC3s state reprogramming toward a highly pro-inflammatory phenotype in mouse AAs. These data provide novel insights into the disease-specific reprogramming of DC subsets and their contributions in AAs.

### 2.5 Modeling of activated cDC2s, DC3s and mregDCs interactions with other cells

**Figure 5:**
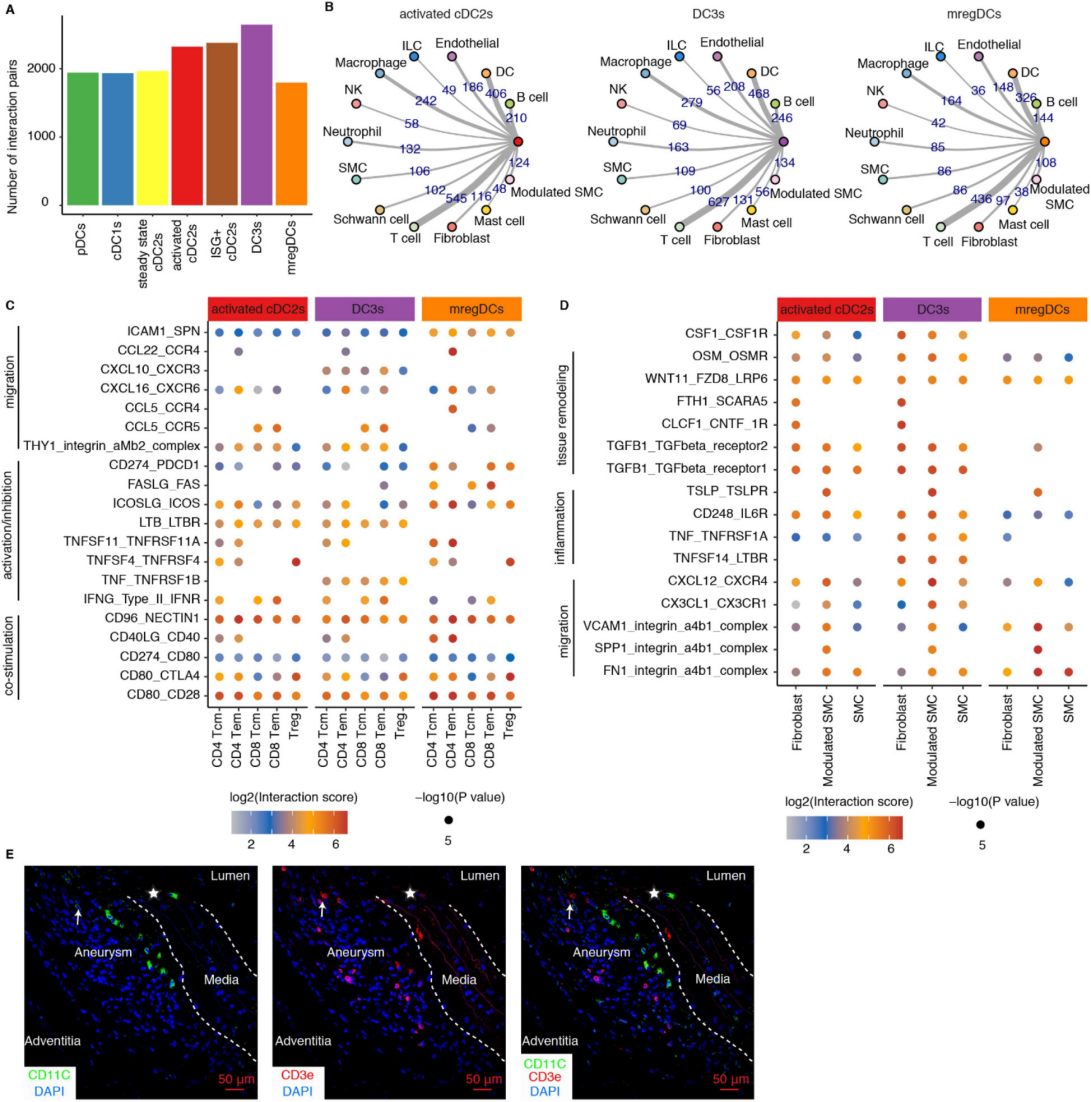
Cell-cell communication modeling reveals cell-cell interaction networks across different mouse AAs. **(A)** Significant interaction pairs between different DC subtypes and other immune and non-immune cells. **(B)** Circular network studies illustrate prominent interactions between activated cDC2s, DC3s and mregDCs with other immune and non-immune cell types. The thickness of edges represents the number of statistically significant interactions between each pair of cell types. EC, endothelial cells; ILC, innate lymphoid cell; NK, natural killer cell; SMC, smooth muscle cell. **(C)** Dot plot depicts representative ligand-receptor interactions between activated cDC2s, DC3s and mregDCs and T cell subtypes in mouse AAs. **(D)** Dot plot depicts representative ligand-receptor interactions between activated cDC2s, DC3s and mregDCs and vascular wall cell subtypes in mouse AAs. *X*-axis denotes different cell types, and the *y*-axis represents ligand-receptor pairs. Dot size represents the statistical *p* value. Color intensities reflect interaction scores. Tcm, central memory T cell; Tem, effector memory T cell; Treg, regulatory T cell. **(E)** Representative images showing the distribution and potential interaction of DCs with T cells in Ang-II-induced AA lesions in mice. Arrows indicate potential interactions of closely neighboring DCs with T cells. Asterisks represent disrupted elastic fibers.

Further, to elucidate the potential mechanisms by which activated cDC2s, DC3s, and mregDCs contribute to the progression of mouse AAs, we performed cell-cell communication modeling analyses using CellPhoneDB to predict potential interactions between DCs and other cell types within the AAs. Restricting our analysis to statistically significant ligand-receptor pairs revealed extensive interactions across DC subtypes, with DC3s exhibiting the highest number of interactions with other cell types (Figure 5A). Predominant interactions involved distinct DC subtype interaction with T cells, other DCs, macrophages, B cells, and endothelial cells (Figure 5B), suggesting their critical role in bridging innate and adaptive immune responses in mouse AAs. Given the critical function of DCs in modulating T cell immune responses, we further examined potential interactions between activated cDC2s, DC3s, mregDCs, and T cells. Our findings revealed that all three DC subtypes promote T cell activation and migration within mouse AAs (Figure 5B). Notably, cDC2s and DC3s displayed similar interaction profiles, whereas mregDCs exhibited distinct patterns, implying their divergent functional roles in AA pathogenesis. Specifically, mregDCs showed strong immunosuppressive interactions with T cells, mediated by ligand-receptor pairs such as CD274-PDCD1, FASLG-FAS, and ICOSLG-ICOS. In contrast, DC3s were distinguished from activated cDC2s and mregDCs by unique TNF-TNFRSF1B and CXCL10-CXCR3 (Figure 5C). Additionally, SMCs and fibroblasts, critical for maintaining vascular structure, were analyzed for their interactions with DCs in mouse AAs (Figure 5D). Our data indicated that these vascular wall cells, particularly modulated SMCs, significantly enhance DCs migration and inflammation responses. Furthermore, several interactions associated with vascular remodeling were observed between vascular wall cells and activated cDC2s and DC3s, highlighting the reciprocal communication between DCs and vascular wall cells as a key contributor to AA pathobiology in mice. Furthermore, we found closely neighboring DCs with T cells in mouse AA lesions, suggesting the potential interactions between DCs and T cells (Figure 5E).

### 2.6 scRNA-seq reveals a common DC landscape in distinct human AAs

**Figure 6:**
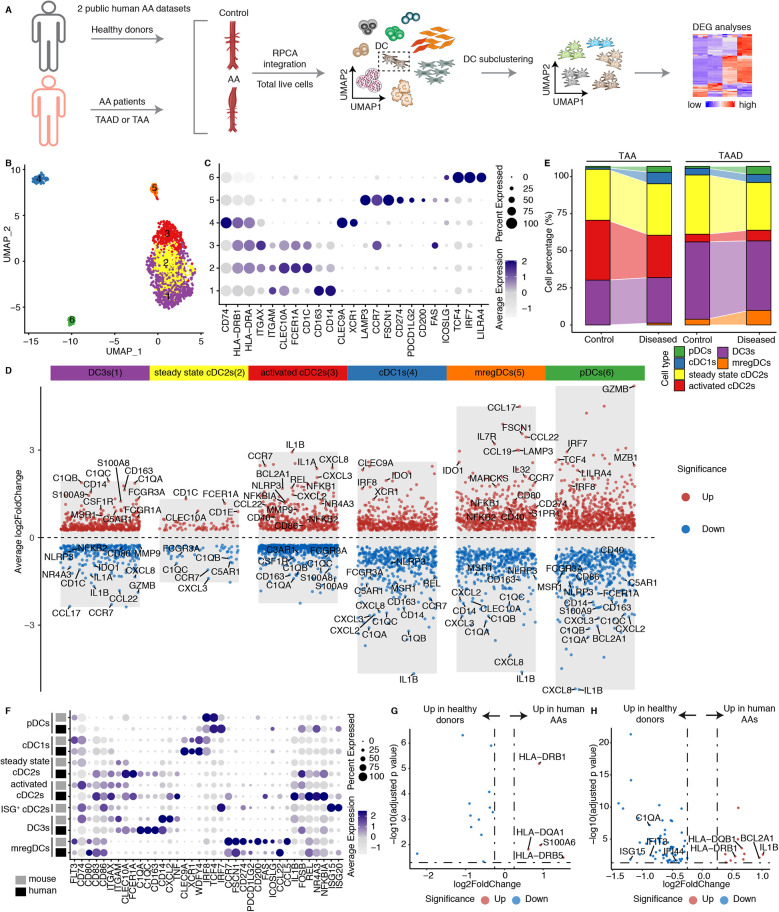
DC subtypes in human AAs. **(A)** Two public human datasets including both healthy donors and AA patients were used for scRNA-seq integration. TAA, thoracic aortic aneurysm; TAAD, Acute type aortic dissection. **(B)** UMAP visualization of the distribution of DC sub-clusters in human AA datasets. **(C)** Dotplot displays the cell type-specific and highly expressed genes in each DC cluster. **(D)** Volcano plot illustrates the cell type- specific and highly expressed genes across different DC subtypes in humans. **(E)** The cell composition of different DC subtypes between healthy donors and diseased AA patients in different human datasets. TAA, thoracic aortic aneurysm; TAAD, Acute type aortic dissection. **(F)** Dotplot showing the cell type or function associated genes across different DC subtypes between mouse and human. The cell definitions of each mouse and human datasets were used for analysis. **(G)** Volcano plot displays the differently expressed genes of activated cDC2s between healthy donors and diseased AA patients. **(H)** Volcano plot displays the differentially expressed genes of DC3s in healthy donors vs. diseased AA patients.

We integrated scRNA-seq from human AA datasets (Figure 6A; Supplementary Table S2). DCs were extracted and further clustered into 6 clusters (Figure 6B). Cells were identified based on lineage-specific cell markers (Figure 6C). Clusters 1, 2, and 3 exhibited high expression of *ITGAX*, *ITGAM*, *CLEC10A* and *FCER1A*, representing cDC2s. Cluster 4 exclusively expressed *CLEC9A* and *XCR1*, i.e., markers for cDC1s. Cluster 5 was defined by high expression of *LAMP3*, *CCR7*, *FSCN1*, and *CD274*, suggesting the presence of mregDCs in human AAs. Cluster 6 was characterized by elevated levels of *TCF4*, *IRF7*, and *LILRA4*, together with the low expression levels of *CD74* and MHC molecules, representing pDCs. We therefore compared the DEGs across different cDC2 clusters. Cluster 1 showed significantly higher expression of *CD163* and *CD14* (Figure 6D), which were established markers of inflammatory DC3s (20, 34). Additionally, cluster 1 displayed upregulation of complement constituents, activation and inflammation-associated genes consistent with the reported inflammatory DC3 phenotype. Similarly, cluster 3 also displayed elevated expression of inflammation-related genes, but downregulated *CD163*, *CD14* and complement-associated genes, suggesting activated cDC2 cells (Figure 6D). In contrast, cluster 2 lacked expression of inflammatory DC3s and activated cDC2s phenotype-related genes, suggesting steady state cDC2s population (Figure 6D). We observed an increased number of DCs in human AAs. However, no significant compositional differences were detected compared to healthy aortas (Supplementary Extended Figure 6). The pro-inflammatory activated cDC2s and DC3s were the primary DC subtypes in human AAs. However, unlike the pronounced increase in activated cDC2s and inflammatory DC3s observed in mouse AAs, these subsets did not exhibit a numerical increase in human AAs compared to healthy donors (Figure 6E). Furthermore, in contrast to aortic dissection, TAAs exhibited a significantly reduced abundance of mregDCs. To translate DC subtypes from mouse to human, we compared the cell type-specific and highly expressed genes of each subtype in mice and human AA tissues (Figure 6F). The large majority of genes were similar between mouse and human DC subtypes, though notable variability was observed in inflammatory DC3s and mregDCs subsets probably reflecting different functional characteristics and stages between defined experimental AA mouse models vs. the more heterogenous human AAs as observed in clinical practice (Figure 6F). Moreover, the diseased immune environment in human AAs did not significantly alter the gene expression profiles of activated cDC2s and inflammatory DC3s, as only limited MHC molecules upregulation in human AAs were observed when compared to their counterpart in their healthy counterparts (Figures 6G,H).

## 3 Discussion

**Figure 7:**
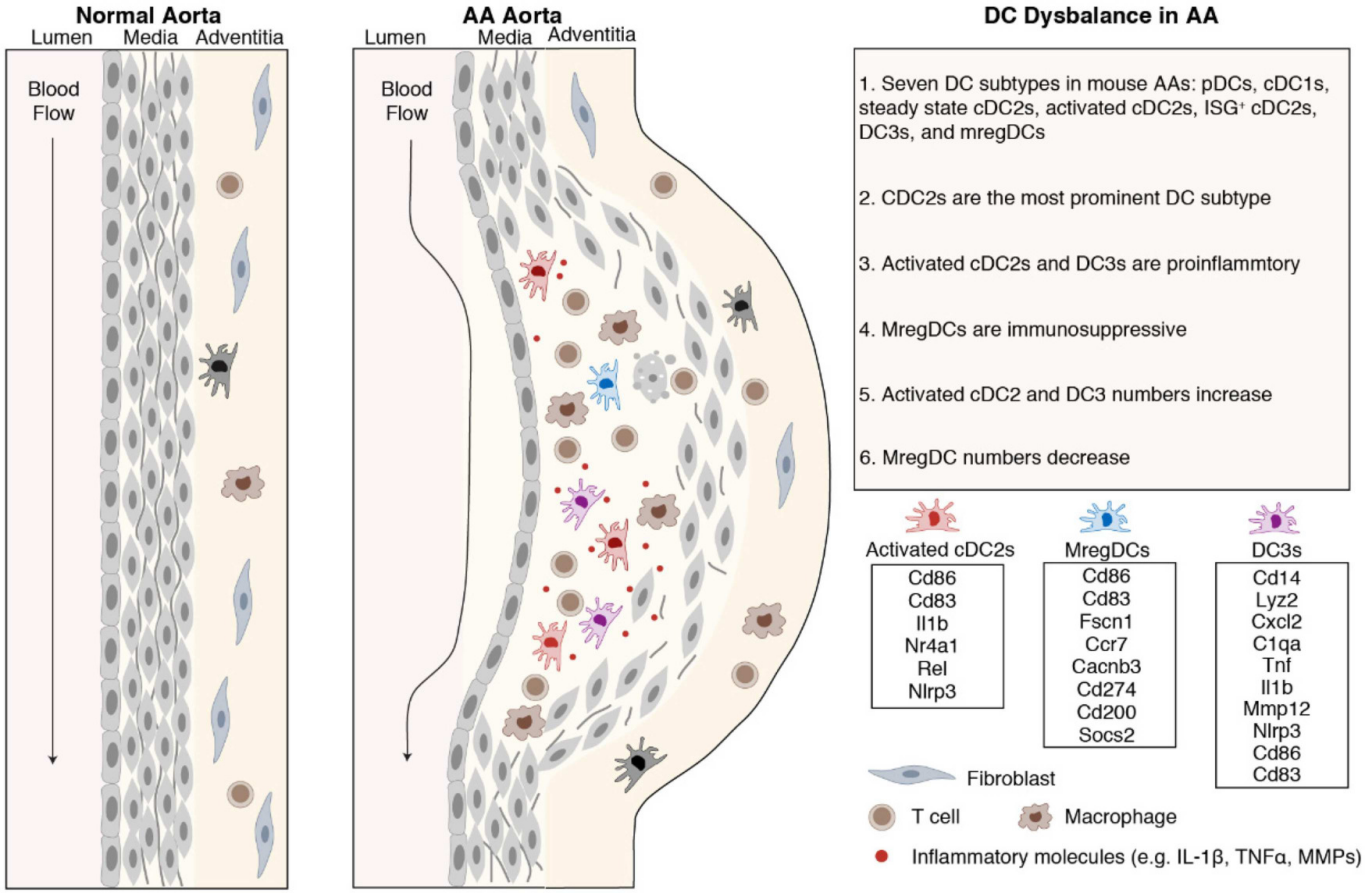
Dysbalance of proinflammatory vs. immunosuppressive DCs in mouse and human AAs. DCs bridge innate and adaptive immune responses. cDC2s were the predominant DC subtype in mouse and human AAs. Activated cDC2s and DC3s show proinflammatory characteristics while mregDCs show immunosuppressive properties.

Using scRNA-seq combined with pan-species integration and cell-cell interaction modeling, our data reported above can be summarized as follows: mouse and human AA DC subtype transcript mapping showed that the dominant DC sublineage consisted of several cDC2 subtypes followed by mregDCs, cDC1s and pDCs; the majority of DCs were highly proinflammatory while mregDCs showed strong immunosuppressive transcript repertoires; activated cDC2s and DC3s appeared to be trained in the AA tissue environment to obtain their respective individual phenotypes observed here; importantly, mregDCs showed a marked numerical reduction when compared to control donor aortas. We conclude that both the numerical composition and the DC training programs uncover a potentially pathophysiology-relevant dysbalance between pro-inflammatory vs. anti-inflammatory/immunosuppresive DCs as drivers of AA progression (Figure 7).

The biological functions of DCs are being comprehensively studied in three clinically important diseases to develop DC-targeted therapeutics: cancer, autoimmune diseases and multiple chronic inflammatory diseases including atherosclerosis (15, 22, 24, 35). In cancer, cancer antigen presentation by DCs is a critical process to enhance cytotoxic CD8^+^ T cells to target specific cancers traits. Several DC-based therapy strategies have been developed for cancer treatment. Moreover, clinical trials have broadly demonstrated the safety and efficacy of DC vaccination in inducing anticancer immune responses (23). Conversely, in autoimmune and chronic inflammatory diseases, inducing immune tolerance through immunosuppressive tolerogenic DCs represent a promising approach to mitigate aberrant inflammatory immune responses (22, 35). Preclinical studies in mouse models of type 1 diabetes, multiple sclerosis, and rheumatoid arthritis have validated the efficacy of treatment approaches using tolerogenic DCs as well as disease antigen-specific tolerogenic DCs in suppressing pathogenic T cell immune responses and promoting immunosuppressive regulatory T cell differentiation (22, 35). Such studies exploit the unique characteristics of DC subtypes to present antigen to T cells including CD4^+^ helper and CD8^+^ cytotoxic T cells to initiate various T cell immune responses to improve clinical outcomes. Although the number of studies investigating DCs in AAs remain limited compared with those in cancer or autoimmune diseases, several pioneering studies have indicated their important role in AA progression. Depletion of CD11c^+^ antigen-presenting cells markedly suppressed experimental AA formation and growth, which was accompanied by reduced circulating effector T cells (36). More recently, Yuan et al. identified that IRF8-driven cDC1 differentiation enhances CD8^+^ T-cell activation, thereby exacerbating AA development (37). These findings indicate that DCs serve as upstream regulators amplifying vascular inflammation via cytotoxic CD8^+^ T-cells. Consistently with these observations, direct genetic evidence has shown that CD8^+^ T-cell deficiency significantly protects mice from AA, whereas adoptive transfer of wild-type CD8^+^ T cells or administration of exogenous IFN-*γ* restores aneurysm formation, confirming a causal contribution of cytotoxic CD8^+^ cells to disease progression (38). In line with these findings, our study supports the concept that DC-based interventions can mitigate aneurysm progression by modulating CD8^+^ T-cell activation and cytotoxicity and may contribute to restoration of vascular immune homeostasis. Nevertheless, the specific roles of distinct DC subsets, especially cDC2s, DC3s and mregDCs in AAs remain incompletely understood, warranting further mechanistic investigation. As we learn more about the impacts of individual DC subtypes for AA growth through future studies, the usage of DCs as immune response-related therapeutics in AA will become more apparent for this deadly disease for which more effective preventive approaches are needed.

Due to the limited accessibility of human AA scRNA-seq datasets, the compositional changes of DC subtypes between control donors and patients with AAs appeared to suggest discrepancies compared with the mouse results. Yet, we identified most DC subtypes in the murine AA models, except for ISG^+^ cDC2s, which may reflect species-specific differences or result from the limited number of DCs captured in the available human datasets. Moreover, DC3s and mregDCs showed variance at the expression levels of key functional genes between mice and humans. These discrepancies may arise from species-specific immune microenvironments, distinct pathological stages of AA, or differences in tissue sampling (e.g., ascending vs. abdominal aorta). Further studies employing large-scale, parallel profiling of human and murine samples with more other methods will be necessary to clarify whether these differences stem from intrinsic interspecies variation or experimental heterogeneity.

Our findings indicate the imbalance of DC subsets within AAs. However, the factors contributing to this imbalance remain to be fully elucidated. Based on our results and previous studies, we hypothesize that multiple microenvironmental factors in the aneurysmal lesion, such as oxidized lipids (39), ECM-derived damage-associated molecular patterns (40), pro-inflammatory cytokines (IFN-γ, TNF-α, IL-1β) (41), and metabolic stress (lactate/cholesterol accumulation) (42, 43), may jointly promote DC maturation, activation, differentiation and migration. These signals may collectively drive the expansion of pro-inflammatory DC subsets and the concomitant reduction of immunoregulatory mregDCs, thereby fostering a sustained inflammatory milieu that contributes to AA progression.

In summary, our current data show that DCs undergo training to become highly proinflammatory. Together with a decrease in mregDCs our data reveal a disease-promoting dysbalance. DC mapping in AA thus can be considered as a blueprint for the further study of DCs in AA to develop therapeutics similar to cancer, autoimmune diseases, and cardiovascular diseases, including atherosclerosis and atherosclerosis-based AAs (44). We suggest that the tools developed in cancer and autoimmune disease research may be applicable to develop DC-based therapeutic to prevent AA progression to attenuate complications of AAs such as dissection of the arterial wall, inhibiting enzymes including proteinases known to be involved in AA complications and by targeting a variety of inflammatory cytokines and mediators of CD8^+^ cytotoxic T cells.

## 4 Materials and methods

### 4.1 Mouse AAs scRNA-seq data collection and integration

All the mouse AAs scRNA-seq involved in the current study were downloaded publicly available scRNA-seq datasets from Gene expression omnibus database (Supplementary Table S1). These datasets included three different mouse model and explain as follows: (i) scRNA-seq was performed on infrarenal abdominal aortas from C57BL/6J mice after perivascular CaCl2 treatment to induce AAs. Infrarenal abdominal aortas were collected 4 days after AA induction and processed for sequencing (GSE164678) (25); (ii) C57BL/6J mice were treated with periadventitial elastase incubation to induce AAs. Infrarenal abdominal aortas were collected after 7 and 14 days post elastase treatment and used for scRNA-seq analysis (GSE152583) (26); (iii) Apoe^−/−^ mice were treated with 4-week angiotensin II to induce mouse AAs. Mouse thoracic and abdominal aortas were used for further scRNA-seq study (GSE221789) (27); (v) Apoe^−/−^ mice were constantly infused with angiotensin II subcutaneously for 4 weeks to induce mouse AAs initiation. AA samples were categorized into 5 different types based on the progression and severity of disease, including abdominal dilated aorta without dissection, single aortic aneurysm without dissection, aortic dissection/intramural haematoma withour aneurysm, single aortic aneurysm with dissection/intramural haematoma, multiple distinct aortic aneurysms with dissection/intramural haematoma. Combined with normal aortas, samples were used for scRNA-seq analysis, respectively (GSE239620) (28).

All the above data were loaded into R and integrated using the Seurat package (version 4.3.0). Low quality cells and cell doublets were filtered out based on the criteria of the number of genes detected per cell and the average expression level of mitochondrial genes in each study, otherwise cells were retained by limiting the number of genes detected in each cell to more than 200 and less than 4,500, and the average expression level of mitochondrial genes was less than 10%. After quality control, genes were normalized across cells and the top 2,000 highly variable genes were identified in each sample. These highly variable genes were scaled and further used for principal component analysis (PCA). Anchors were identified by RPCA method in Seurat package and representative samples from each dataset were selected as references for integration. The top 30 significant principal components (PCs) were used to construct the shared nearest-neighbor (SNN) graph and the top 30 significant PCs were used for UMAP visualization. Cell clusters were defined by setting the resolution to 0.5 for total cell analysis. The major cell types were defined by using cell type specific markers. We adopted the UMAP visualization, iLISI and cLISI scores before and after batch effect correction to evaluate the batch effect during sample integration. iLISI and cLISI scores were calculated using the “compute_lisi” function from the lisi package (version 1.0). A higher iLISI score and a lower cLISI score indicate more successful batch effect removal.

### 4.2 Mouse DCs integration and sub-clustering

For further functional diversity analysis of DCs, we first extracted all total DCs from the above integrated dataset based on the specific gene markers of DCs, like *Flt3* and *Cd74*. These extracted DCs were then re-clustered by using the RPCA method as we described above. The top 20 significant PCs were used to construct the SNN graph and the top 20 significant PCs were used for UMAP visualization. Cell clusters were defined by setting the resolution to 0.7. DCs clusters were recognized by using the reported specific gene markers (18, 21). The gene expression profiles of each cluster were investigated by comparing the DEGs of each DC subtypes with “FindAllMarkers” or “FindMarkers” functions in Seurat package.

To compare the gene expression profiles of DCs in diseased mouse AAs with those from homeostatic splenocyte DCs, which contains well-defined DC subtype populations (https://doi.org/10.6084/m9.figshare.22232056.v1) (18). We further integrated the mouse AA DCs with splenocyte DCs. The top 25 significant PCs were used to construct the SNN graph and the top 25 significant PCs were used for UMAP visualization. Cell clusters were defined by setting the resolution to 0.8. The difference of gene expression profiles between hemostatic and diseased conditions were investigated by compared the DEGs of well-defined DC subtypes between two different conditions.

### 4.3 Pathway enrichment analysis

The significant DEGs identified for each cluster using the “FindAllMarkers” or “FindMarkers” functions in the Seurat package were utilized as input genes for Gene Ontology pathway enrichment analysis, performed with the clusterProfiler package (version 4.4.4). *P* values for enriched pathways were adjusted using Bonferroni correction. Only pathways with an adjusted *p* value less than 0.01 and containing more than 10 enriched genes were selected for visualization.

### 4.4 Gene signature module score analysis

The gene signature module scores across different DC subtypes in mouse AAs scRNA-seq data were calculated by “AddModuleScore_UCell” function in the UCell package (version 2.2.0). The following genes were used to calculate anti-apoptotic signature scores: *Bcl2*, *Bcl2l1*, *Mcl1*, *Bcl2l12*, *Bcl2l13*, *Birc2*, *Birc3*, *Birc5*, *Birc6*, *Xiap*, *Bcl2a1a*, *Bcl2a1b*, *Bcl2a1c*, and *Bcl2a1d*. Meanwhile, genes including *Cd40*, *Cd80*, *Cd86*, and *Cd83* were used to calculate the co-stimulatory gene signature scores (19). *Cd274*, *Pdcd1lg2*, *Cd200*, *Fas*, *Adh1a2*, *Socs1*, and *Socs2* were selected to calculate the immunosuppressive signature scores of DCs. Genes including *Ccr7*, *Myo1g*, *Cxcl16*, *Adam8*, *Icam1*, *Fscn1*, *Marcks*, and *Marcksl1* were used to evaluate the DCs migration capacity signature scores. MHC molecule gene signature scores were calculated by including *H2-K1*, *H2-Oa*, *H2-DMa*, *H2-DMb2*, *H2-DMb1*, *H2-Ob*, *H2-Ab1*, *H2-Aa*, *H2-Eb1*, *H2-D1*, *H2-Q4*, *H2-Q6*, *H2-Q7*, *H2-Q10*, *H2-T23*, *H2-T22*, *H2-M3*, *H2-Eb2*, H2-T24, *H2-M2*, *H2-M9*, *H2-M5*, *H2-Q1*, and *H2-Q2*.

### 4.5 Cell-cell communication analysis

To explore the differences of cell-cell interactions between different mouse DC subtypes and other cell types. The integrated mouse transcriptomic genes were converted to human homologous genes via “getLDS” function in biomaRt package (version 2.62.1) based on the mouse and human ensemble database. CellphoneDB (version 5) was adopted to predict the significant interaction across different cell types in Python. The output interaction scores, means, and *p* values files were combined to define the significant interactions. The interactions with interaction score more than 0 and *p* values less than 0.05 were considered as significant interactions. *P* values less than 0.000001 were re-set to 0.000001 for better data visualization.

### 4.6 Human AAs scRNA-seq data collection and integration

The publicly available human scRNA-seq data associated with AAs injury were download from Gene expression omnibus for analysis (Supplementary Table S2). These datasets include: (i) Ascending aortic wall tissue from 6 acute type A aortic dissection patients and controls from 3 heart transplant donors were collected and used for scRNA-seq analysis (GSE213740) (45); (ii) Ascending aortic tissues from 8 patients with ascending thoracic aortic aneurysm and 3 controls were obtained and categorized by using scRNA-seq (GSE155468) (46). The procedures for human data integration were same to mouse studies as described above. Briefly, all data were loaded into R and processed by Seurat (version 4.3.0) package. Low-quality cells and cell doublets in each sample were filtered out by limiting the number of genes detected in each cell to more than 200 and less than 5,000, and the average expression level of mitochondrial genes was less than 20. The data were then normalized and log transformed to remove the confounding of different sequence depths. 2,000 highly variable genes among cells were identified in each sample, which were scaled and further used for PCA analysis. Anchors were identified by RPCA method in Seurat package and representative samples from each dataset were selected as references for integration. The top 10 significant PCs were used to construct the SNN graph and the top 10 significant PCs were used for UMAP visualization. Cell clusters were defined by setting the resolution to 0.3 for total cell analysis. The major cell types were identified for further sub-clustering using cell type specific markers.

### 4.7 Human DCs integration and sub-clustering

DCs were extracted and sub-clustered using the procedures described above. The top 30 significant PCs were used to construct the SNN graph and the same top 30 significant PCs were employed for UMAP visualization. Cell clusters were defined by setting the resolution to 0.3. DC clusters were identified using DEGs and reported cell markers (19). The percentage of DC subtypes in total DCs were calculated as: number of DC subtypes in each sample ÷ number of total DCs in each sample × 100%. To assess the batch effect correction after integration, we employed the iLISI score as described in mouse study as well.

### 4.8 Histology and immunofluorescence

Animal procedures were approved by the Ethics Committee of Sun Yat-sen University according to the guidelines of the local Animal Use and Care Committee and the National Animal Welfare Laws. Paraffin-embedded Ang-II induced mouse aorta sections were first deparaffinized and rehydrated using standard protocols. Tissue sections were stained with hematoxylin for 10 s, followed by differentiation in 1% acid ethanol for 5 s. Bluing was achieved by rinsing the slides under running tap water for 5 min. The sections were subsequently stained with eosin for 4 min, dehydrated in absolute ethanol for 30 s, and air-dried. The slides were mounted with neutral resin and imaged under a light microscope.

Paraffin-embedded AAs sections used for Immunofluorescence staining were first deparaffinized and rehydrated using standard protocols. Antigen retrieval was performed by heating sections in EDTA solution (pH 8.0) in a microwave oven. To minimize non-specific binding, sections were blocked with 5% BSA supplemented with anti-CD16/32 antibody for 1 h at room temperature. Subsequently, sections were incubated overnight at 4℃ with primary antibodies diluted in 5% BSA solution. The following primary antibodies were used for staining: anti-CD11c (clone N418, 1:50, Invitrogen), anti-CD14 (clone SC69-02, 1:200, Invitrogen), polyclonal anti-CD3e (Abcam, 1:300), anti-Fscn1 (clone EP5902,1:200, Abcam). Following incubation, sections were stained with appropriate secondary antibodies. To mitigate potential autofluorescence, sections were treated with an autofluorescence quencher solution (Servicebio) in accordance with the manufacture's protocol. Stained sections were visualized using a Zeiss LSM880 microscope. All images were prepared as TIF files by Fiji (ImageJ, National Institutes of Health) and exported into Adobe Illustrator CS6 for figure arrangements.

### 4.9 Statistical analysis

Data were analyzed in R by using stats package (version 4.2.2). The data distribution was firstly tested by Shapiro–Wilk test (“shapiro.test” function in stats package). If datasets did not follow Gaussian distribution, the difference between two groups was compared by two-sided Wilcoxon rank-sum test (“Wilcox.test” function in stats package) and difference between three or more groups was analyzed by non-parametric Kruskal–Wallis *H* test (“Kruskal.test” function in stats package). Differences were considered significant at a two-tailed *P* value <0.05.

## Data Availability

Publicly available datasets were analyzed in this study. This data can be found here: GEO database (https://www.ncbi.nlm.nih.gov/geo/), accession numbers: GSE164678, GSE152583, GSE221789, GSE239620, GSE213740 and GSE155468. The well-defined DC subsets in homeostatic spleen DCs are available for download at the following link: https://doi.org/10.6084/m9.figshare.22232056.v1.
