# Supplementary material for: Single cell RNA sequencing reveals a dysbalance of proinflammatory vs. immunosuppressive dendritic cells in mouse and human aortic aneurysms
Source: Front Cardiovasc Med. 2025 Nov 19;12:1713030. doi: 10.3389/fcvm.2025.1713030 (PMC12672890; doi:10.3389/fcvm.2025.1713030)
Supplement: Supplementary file 1 [file Datasheet1.pdf]

Single cell RNA Sequencing Reveals a Dysbalance of Proinflammatory versus Immunosuppressive Dendritic Cells in Mouse and Human Aortic Aneurysms

Yi Ran et al.,

Supplementary Material

1 Supplementary Data

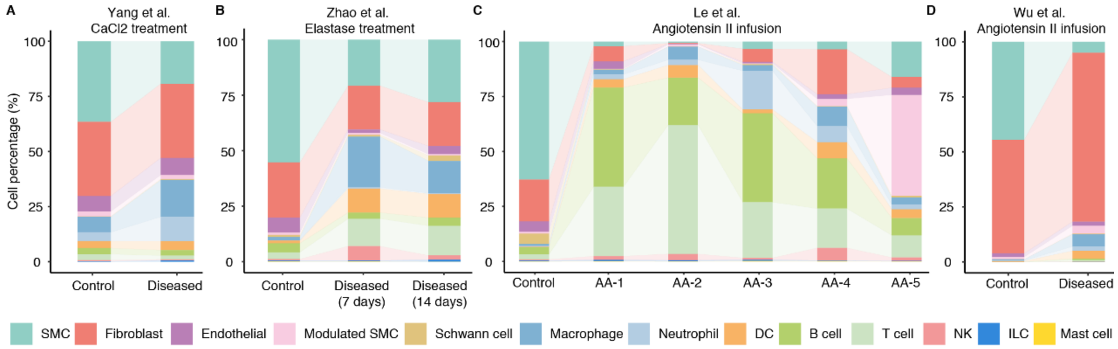

Extended Figure 1. Cell percentage changes across 4 different mouse AA studies. The changes of major cell subtypes proportion in Yang et al. (**A**), Zhao et al. (**B**), Le et al. (**C**) and Wu et al. (**D**) studies. AA-1 to AA-5 in Le et al. study represent different types of mouse AAs. AA-1: abdominal dilated aorta without dissection; AA-2: single aortic abdominal aneurysm without dissection; AA-3: abdominal aortic dissection/intramural haematoma without aneurysm; AA-4: single abdominal aortic aneurysm with dissection/intramural haematoma; AA-5: multiple distinct abdominal aortic aneurysms with dissection/intramural haematomas. SMC: smooth muscle cell; NK: natural killer cell; ILC: innate lymphoid cell.

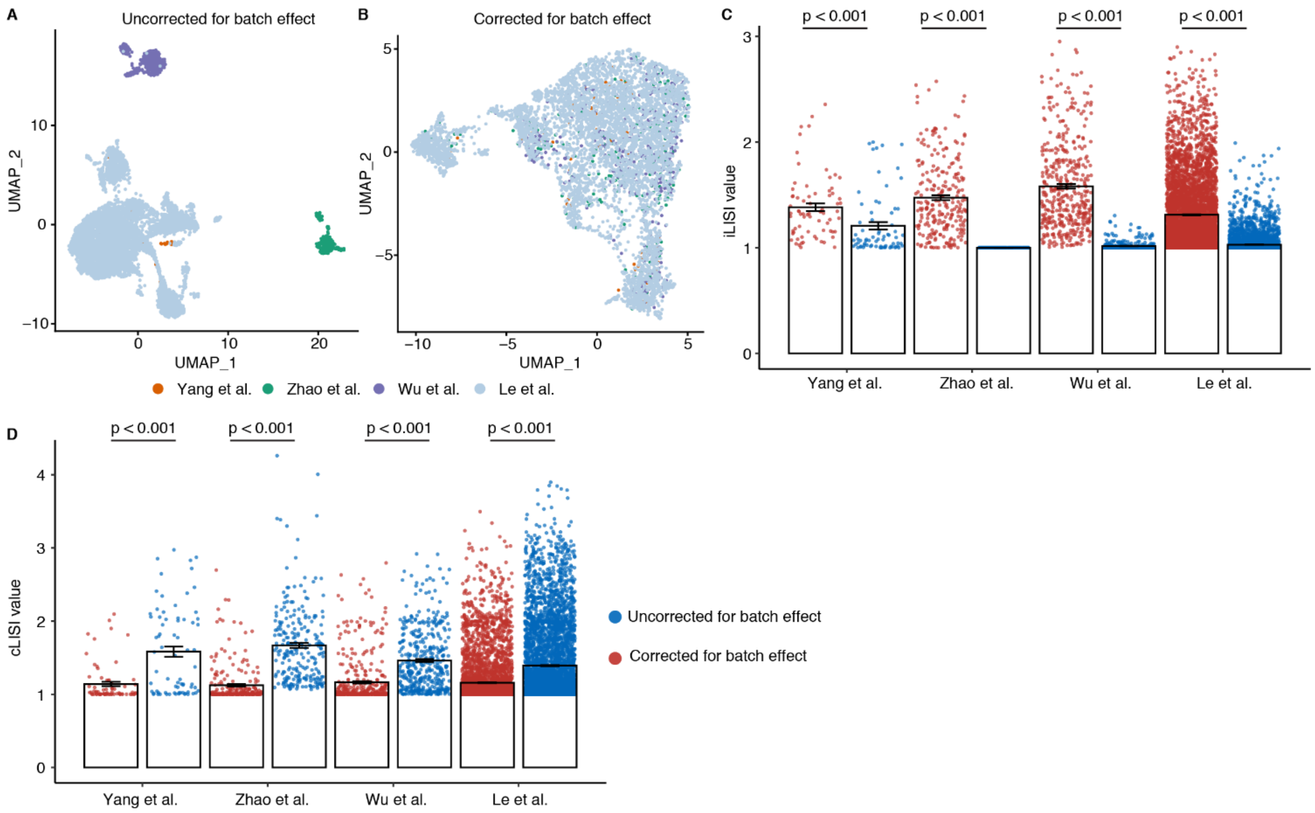

Extended Figure 2. Batch effect correction approach after multi-scRNA-seq dataset integration. A. UMAP visualization of cell distribution without batch effect correction of each dataset. **B.** UMAP visualization of cell distribution after batch effect correction of each dataset. **C.** Integration LISI (iLISI) value to measure the mixing of batches before and after batch effect correction. Each dot represents one single cell. Higher iLISI scores indicate better removal of batch effects, as cells from different datasets are more intermixed within local neighborhoods. **D.** Cell-type LISI (cLISI) value to measure the purity of cell-type clusters before and after batch effect correction. Each dot represents one single cell. Lower cLISI scores are desirable, indicating that neighborhoods are dominated by a single cell type and biological identity is preserved. The Wilcoxon rank sum test was used to compare the differences in iLISI values before and after correction for batch effect.

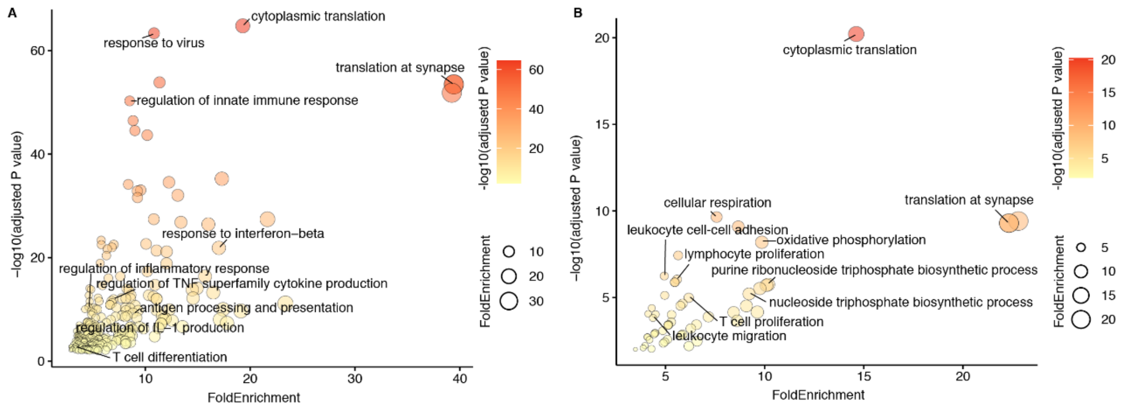

Extended Figure 3. Pathway enrichment analyses of ISG^{+} cDC2s (A) versus quiescent/steady state cDC2s in mouse AAs (B).

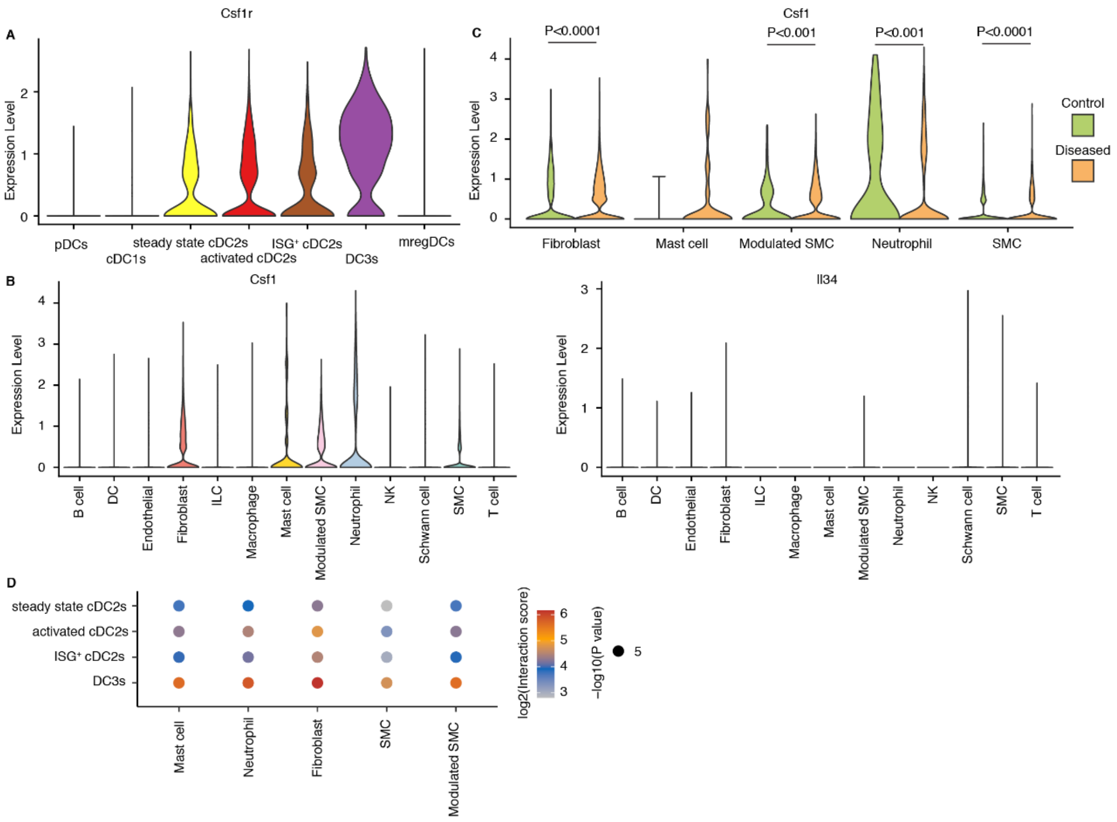

Extended Figure 4. Mouse AAs disease site promotes DC differentiation via Csf1r signaling. A. Violin plot showing the gene expression level of Csf1r across distinct DC subtypes. **B.** Violin plot illustrating the expression levels of Csf1 and Il34, the ligands for Csf1r, across various cell subtypes. **C.** Comparative analysis of Csf1 gene expression between control and diseased aortas across different cell subtypes, with statistical significance assessed using the Wilcoxon rank-sum test. Comparative analysis of Csf1 gene expression between control and diseased aortas across different cell subtypes, with statistical significance assessed using the Wilcoxon rank-sum test. **D.** Dot plot visualizing the interactions between CSF1 and CSF1R among DC subtypes and Csf1-expressing cell subtypes. The x-axis denotes different cell types, and the y-axis represents ligand-receptor pairs. Dot size represents the statistical p value. Color intensity reflects interaction score.

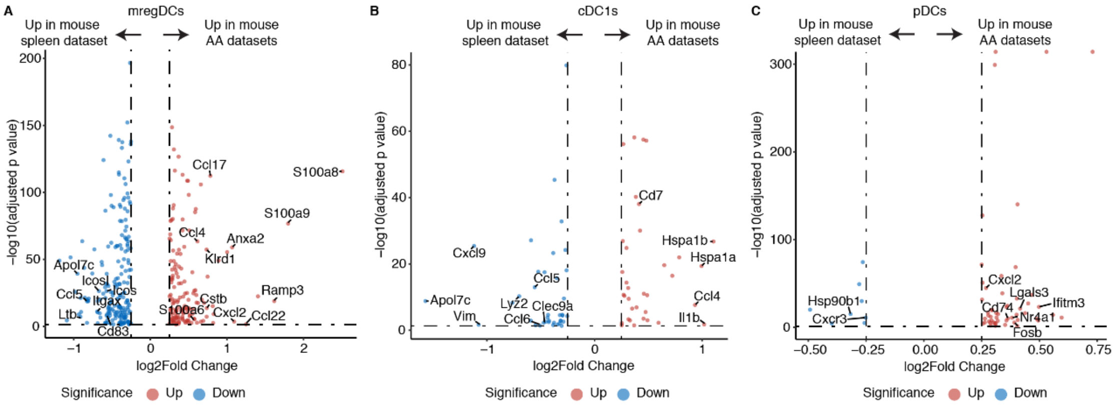

Extended Figure 5. Differentially expressed genes in mouse AA datasets and mouse spleen datasets in mregDCs (A), cDC1s (B) and pDCs (C). Each dot represents a statistically significant differentially expressed gene. The x-axis indicates the log2-transformed fold change, and the y-axis represents the -log10-transformed adjusted p-value. Only differentially expressed genes meeting significance thresholds are displayed.

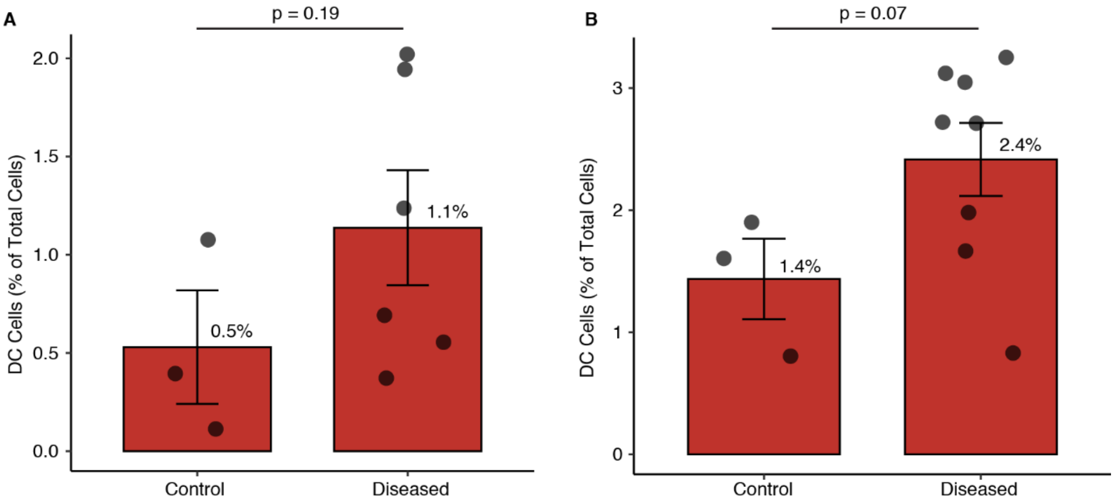

Extended Figure 6. Changes in DC percentages in two different human AA studies. Proportions of DCs in human acute type aortic dissection (**A**) and ascending thoracic aortic aneurysm (**B**) studies. A t-test was used to assess difference between groups. Each dot represents an individual sample, with the number above each bar indicating the mean percentage for the respective group.

Supplementary Table 1. Information on mouse datasets for multi-sample scRNA-seq integration.

Data set	Platform	Treatment	Tissue	Cell type	Mouse strain	Number	GEO	PMID

1	Yang et al.	Chromium Single cell 3' Reagent Kits v3	perivascularly treated with 0.5 M CaCl2 for 10 minutes followed by PBS for 5 minutes.perivascularly treated with 0.5 M CaCl2 for 10 minutes followed by PBS for 5 minutes	Infrarenal abdominal aortas	All cells	C57BL/6J	pooled sample from 4 mice	GSE164678	PMID: 33472403
2	Zhao et al.	Chromium Single cell 3' Reagent Kits v2	elastase-induced AAA. Aortas were collected at days 7 and 14 post periadventitial elastase treament.	Infrarenal abdominal aortas	All cells	C57BL/6J	pooled sample from 5 mice	GSE152583	PMID: 32678909
3	Wu et al.	Chromium Single cell 3' Reagent Kits v2 or v3	4 wks Angiotensin II-induction	thoracic and abdominal aorta	All cells	ApoE-deficient	pooled sample from 2-8 mice in different groups	GSE221789	PMID: 36703732
4	Le et al.	Chromium Single cell 3' Reagent Kits v2	4 wks Angiotensin II-induction	abdominal aorta	All cells	ApoE-deficient	pooled sample from 7 mice in different groups	GSE239620	PMID: 38836630

Supplementary Table 2. Information on human datasets for multi-sample scRNA-seq integration.

	Data set	Platform	Tissue	Cell type	Number	GEO	PMID
1	Zhang et al.	Chromium Single cell 3' Reagent Kits v2	Ascending aortic wall	All cells	Ascending aortic wall tissue from 6 sporadic acute type A aortic dissection patients and 3 heart transplant donors	GSE213740	PMID: 36830768
2	Li et al.	Chromium Single cell 3' Reagent Kits v3	ascending aortic tissue	All cells	8 patients with ascending thoracic aortic aneurysm and 3 controls	GSE155468	PMID: 33017217
